# Specific CLK Inhibitors from a Novel Chemotype for Regulation of Alternative Splicing

**DOI:** 10.1016/j.chembiol.2010.11.009

**Published:** 2011-01-28

**Authors:** Oleg Fedorov, Kilian Huber, Andreas Eisenreich, Panagis Filippakopoulos, Oliver King, Alex N. Bullock, Damian Szklarczyk, Lars J. Jensen, Doriano Fabbro, Jörg Trappe, Ursula Rauch, Franz Bracher, Stefan Knapp

**Affiliations:** 1 University of Oxford, Nuffield Department of Clinical Medicine, Structural Genomics Consortium, Old Road Campus Research Building, Oxford OX3 7DQ, UK; 2 Ludwig-Maximilians Universität, Department of Pharmacy-Center for Drug Research, Butenandtstrasse 5-13, 81377 Munich, Germany; 3 Charité-Universitätsmedizin Berlin, Campus Benjamin Franklin, Centrum für Herz-und Kreislaufmedizin, Berlin, Germany; 4 NNF Center for Protein Research, Faculty of Health Sciences, University of Copenhagen, Denmark; 5 Novartis Pharma AG, Klybeckstrasse 141, CH-4002 Basel, Switzerland; 6 University of Oxford, Department of Clinical Pharmacology, Old Road Campus Research Building, Oxford OX3 7DQ, UK

## Abstract

There is a growing recognition of the importance of protein kinases in the control of alternative splicing. To define the underlying regulatory mechanisms, highly selective inhibitors are needed. Here, we report the discovery and characterization of the dichloroindolyl enaminonitrile **KH-CB19**, a potent and highly specific inhibitor of the CDC2-like kinase isoforms 1 and 4 (CLK1/CLK4). Cocrystal structures of **KH-CB19** with CLK1 and CLK3 revealed a non-ATP mimetic binding mode, conformational changes in helix αC and the phosphate binding loop and halogen bonding to the kinase hinge region. **KH-CB19** effectively suppressed phosphorylation of SR (serine/arginine) proteins in cells, consistent with its expected mechanism of action. Chemical inhibition of CLK1/CLK4 generated a unique pattern of splicing factor dephosphorylation and had at low nM concentration a profound effect on splicing of the two tissue factor isoforms flTF (full-length TF) and asHTF (alternatively spliced human TF).

## Highlights

► We report a highly selective nanomolar inhibitor KH-CB19 for the kinases CLK1/4 and DYRK1 ► KH-CB19 cocrystal structures revealed an ATP competitive but not ATP mimetic binding mode ► KH-CB19 formed halogen bonds with the kinase hinge region ► KH-CB19 led to dephosphorylation of SR proteins and effected splicing of TF isoforms in cells

## Introduction

There are about 23,000 protein-coding genes in the human genome. However, the human proteome consists of a far larger number of unique protein sequences. In fact, some 90% of all transcribed genes may undergo alternative splicing and more than 80% may have at least 15% abundance of minor splicing forms (Shi et al., 2008). In many cases, alternative splicing leads to the expression of several protein isoforms with different and sometimes antagonistic functions (Pajares et al., 2007). Notable examples include pro- and antiapoptotic isoforms of Bcl-2 family members (Akgul et al., 2004) and pro- and antiangiogenic forms of VEGFA (Harper and Bates, 2008). This plasticity plays a fundamental role in tissue development and the cellular response to external stimuli, for example in the control of blood clotting (Eisenreich et al., 2009) and insulin action (Jiang et al., 2009). Not surprisingly, the deregulation of alternative splicing has also been linked to numerous human pathologies (Ward and Cooper, 2010).

The regulation of alternative splicing is highly complex. In addition to the essential enzymatic step of RNA breakage and ligation, the spliceosome must recognize the exon and intron boundaries precisely and in a controlled fashion. Not surprisingly, the splicing machinery involves hundreds of auxiliary factors that control splice site selection, spliceosome assembly and the splice reaction (Wahl et al., 2009; Bourgeois et al., 2004). Indeed, the spliceosome alone rivals the ribosome and chromatin remodeling complexes in its complexity (Ritchie et al., 2009). But what distinguishes the spliceosome is its very dynamic nature. During the different stages of the splicing process, dozens of proteins get recruited or dissociated from the spliceosomal complex (Wahl et al., 2009). The availability and posttranslational modification status of these regulatory proteins define the outcome of the splicing reaction and link it to extracellular signaling (Blaustein et al., 2007). One group of proteins regulating the selection of alternatively spliced exonic or intronic premessenger (mRNA) sequences in response to environmental changes are serine/arginine-rich (SR) proteins (Bourgeois et al., 2004). The group name relates to the serine/arginine-rich sequences present in these proteins (Long and Caceres, 2009). The serine residues in these sequence patches are phosphorylated by several protein kinase families, most notably the serine/arginine-rich protein kinases (SRPKs) and the CDC2-like kinase family (CLKs) (Colwill et al., 1996; Gui et al., 1994). The phosphorylation status of SR proteins regulates in turn their cellular localization and activity (Stamm, 2008).

The phosphorylation-dependent signal transduction is a recurrent theme in cell signaling and the control of alternative splicing appears to be no exception. Given the recent success in designing selective kinase inhibitors, several efforts have been made to target CLKs. Muraki et al. (2004) reported a cell permeable benzothiazole compound (**TG003**) with 20 nM and 15 nM potency for CLK1 and CLK4, respectively. However, more comprehensive profiling of this compound revealed strong inhibition of **TG003** for all CLK family members except for CLK3 but also cross reactivity with casein kinase (CK1δ and CK1ɛ), dual-specificity tyrosine phosphorylation-regulated kinase (DYRK1B), Yeast Sps1/Ste20-related kinase (YSK4) and proviral insertion site in Moloney Murine Leukemia Virus (PIM) kinase isoforms (Mott et al., 2009). The latter paper reported also a series of substituted 6-arylquinazolines with low nM potencies inhibiting all CLKs as well as DYRK1A and DYRK1B and the tyrosine kinase EGFR. In addition, a number of nonselective inhibitors have been reported together with the crystal structures of CLK1 and CLK3 (Bullock et al., 2009). However, to date there are still no potent and highly selective CLK inhibitors with the submicromolar cellular activity available that would be required for use in in vivo experiments. Chemical probes with such characteristics may help to decipher the role of CLKs not only in splicing regulation, but also in the control of viral infections (Karlas et al., 2010) as well as cellular metabolism (Rodgers et al., 2010).

Here, we describe a novel class of CLK inhibitors (dichloroindolyl enaminonitriles), with high specificity for CLK1/CLK4 isoforms and a unique binding mode to the kinase hinge region. The lead compound shows single-digit nanomolar activity in modulating alternative splicing in human endothelial cells.

## Results

**Figure 1:**
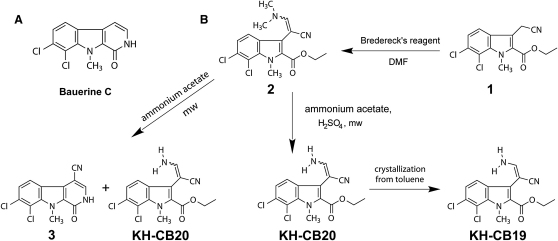
Synthetic Route (A) Lead alkaloid **Bauerine C**. (B) Synthetic route for the preparation of the studied inhibitors.

Natural compounds provide a rich source for novel chemical scaffolds which offer an excellent foundation for rational structure-based design. Recently, we reported a novel class of potent and selective class III histone deacetylase (sirtuin) inhibitors, which are structural hybrids between a common kinase inhibitor scaffold and the β-carboline alkaloid bauerine C (Figure 1A), having a unique 7,8-dichloro substitution pattern (Huber et al., 2010a). Bauerine C was originally isolated from the blue-green alga *Dichothrix baueriana* and has been reported to have antiproliferative as well as antiviral properties (Larsen et al., 1994). In this study, we envisaged to prepare a library of novel bioactive compounds using 4-cyano-bauerine C (**3**), an easy-to-functionalize derivative of the alkaloid bauerine C, as a basis for structural diversification (Figure 1B).

**Figure 2:**
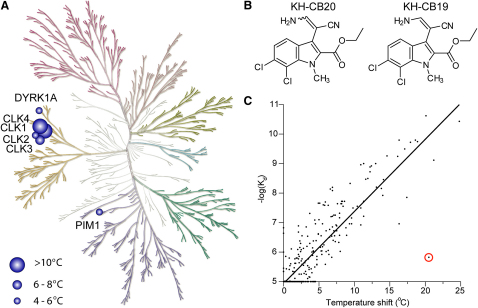
Activity and selectivity of KH-CB19 (A) Binding of **KH-CB19** to kinase catalytic domains as assessed in thermal shift assays against 129 human protein kinases (see Table S1 for screened targets). Targets that showed significant temperature shifts are highlighted by blue spheres. (B) Chemical structures for **KH-CB19** and **KH-CB20**. (C) Correlation between binding constants determined by AMBIT and temperature shift data. The correlation of the linear least-squares fit was 0.95. One outlier is highlighted (red circle) which corresponds to **BIRB-796**, a potent p38 inhibitor. This compound showed only weak association in the AMBIT assay possibly due to slow binding kinetics, but gave a high T_m_ shift as expected for this potent inhibitor. The kinome phylogenetic tree has been used and modified with permission of Cell Signaling Technology.

For the preparation of 4-cyano-bauerine C (**3**) we started from ethyl 3-cyanomethyl-6,7-dichloro-1-methyl-1*H*-indole-2-carboxylate (**1**) (Huber et al., 2010b), which was reacted with Bredereck's reagent (*tert*-butoxy-bis(dimethylamino)methane) to give the tertiary enaminonitrile **2** as a mixture of *E/Z* isomers. This intermediate was then heated with ammonium acetate and glacial acetic acid in a microwave reactor to give 4-cyano-bauerine C (**3**). Both **3** and the intermediate tertiary enaminonitrile **2** were screened against a panel of 106 kinases using a thermal shift assay and showed only interaction with CLK family members (see Table S1 available online). Primary enaminonitrile **KH-CB20** (as an *E/Z* mixture) was originally isolated as a side product in the synthesis of **3** and also screened against the kinase panel. Serendipitously, the kinase assay revealed **KH-CB20** to be a potent and selective inhibitor of CLK1 and the closely related isoform CLK4, with significantly reduced affinity to CLK2 and CLK3 (Figure 2; Table S1). Thus the procedure for the synthesis of **KH-CB20** was optimized. Addition of sulfuric acid to the reaction mixture and shorter reaction time largely prevented cyclisation to 4-cyano-bauerine C (**3**) and led to predominant formation of the primary enaminonitrile **KH-CB20** as a 71:29 mixture of *E*- and *Z*-isomers. Separation of the *E*-isomer **KH-CB19** could be achieved by selective recrystallization from toluene. The pure *E*-isomer **KH-CB19** and the *E/Z*-mixture **KH-CB20** had similar kinase binding activity (Table S1).

**Figure 3:**
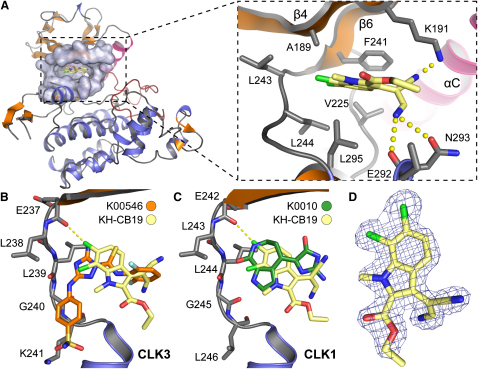
Binding Mode of CLK Inhibitors (A) Overview of the CLK cocrystal structure. The CLK1 catalytic domain is shown as a ribbon diagram and the ATP binding site has been highlighted in surface representation. Details of the interaction of **KH-CB19** with the kinase active site are shown in the detailed view on the right. (B) Superimposition of the CLK3 cocrystal structure with **KH-CB19** and the triazole diamine K00546. (C) Superimposition of CLK1 complexes with **KH-CB19** and hymenialdisine (K0010). (D) Electron density (2Fc-2Fo) map of **KH-CB19** in the CLK1 complex. The map has been contoured using a 2 sigma cutoff.

Direct measurements of kinase inhibition in enzymatic assays revealed low nM potencies. Both **KH-CB20** and **KH-CB19** showed potent inhibition of CLK1 with an IC_50_ of 20 nM, and for the pure isomer **KH-CB19**, almost 100-fold selectivity against the CLK3 isoform (Figure 2 and Table 1). Using temperature shift assays, cross-screening against 129 kinases revealed only strong interaction with CLK family members, in particular CLK1 and CLK4. We were interested to assess how temperature shift data across such a wide and diverse panel of kinases correlate with binding affinities. To do this, we used the large panel of binding data that has been made available by AMBIT (Fabian et al., 2005; Karaman et al., 2008) and temperature shift data that have been published previously by our laboratory (Fedorov et al., 2007). As shown in Figure 2C, the thermal shift data showed good overall correlation (R = 0.95) with published AMBIT binding constants. However, weaker hits identified in temperature shift assays sometimes still correspond to potent inhibitors in enzyme kinetic assays. Unfortunately, this was also the case for DYRK1A which showed a temperature shift of 5.4°C that corresponded to an IC_50_ of 55 ± 6 nM in enzyme kinetic assays (Table 1). To further confirm specificity, **KH-CB19** was profiled against a panel of 71 protein kinases (see Manley et al. [2010] for panel members) using an enzymatic activity assay. No additional kinases from the panel were inhibited confirming the inhibitor selectivity for CLKs. The exceptionally specific activity and unique chemical structure, which does not resemble any known kinase inhibitor, prompted us to determine the crystal structure of **KH-CB19** complexes with both CLK1 and CLK3. In addition, we determined the cocrystal structure of CLK3 with a typical ATP mimetic triazole diamine inhibitor, **K00546** (5-amino-3-[{4-aminosulfonyl}phenylamino]-*N*-2,6-difluorophenyl)-1*H*-1,2,4-triazole-1-carbothiamide), which has been published as a potent CDK1 and CDK2 inhibitor (Lin et al., 2005) (for refinement and data collection statistics, see Table 2). The cocrystal structure with **KH-CB19** revealed that the inhibitor bound to the ATP binding site in CLK1 and CLK3 (Figure 3A). However, due to the lack of hydrogen bond donors or acceptors at the carbocyclic ring, **KH-CB19** did not interact with the hinge region with a canonical ATP mimetic binding mode (Figure 3B). Instead, **KH-CB19** formed a halogen bond with the main chain carbonyl of Glu242. The Cl⋯O distance was 2.9 Å, below the sum of van der Waals radii (3.3 Å) of carbon-bound chlorine and sp^2^-hybridized oxygen. The linear C-Cl⋯O geometry also fulfilled the geometrical criteria for a chlorine halogen bond to the kinase backbone (Voth and Ho, 2007). Interestingly, superimposition with the triazole diamine cocrystal structure revealed one Cl atom in the same position as the primary amine nitrogen that forms a hydrogen bond with the hinge backbone. Similarly, the role and geometry of this halogen bond for **KH-CB19** kinase interaction was evident from superimposition with the CLK1/hymenialdisine (K0010) cocrystal structure (Bullock et al., 2009) (Figure 3C). In both cases, the chlorine atom occupied the position of the hydrogen bond donor of common kinase inhibitors, mimicking the NH_2_ group of ATP. The contribution of halogen bonds to ligand affinity and specificity has not been fully determined and may vary (Bissantz et al., 2010). The second chlorine atom of **KH-CB19** was positioned outside of halogen bond range and formed more common lipophilic interactions. Overall, the inhibitor was well defined by electron density (Figure 3D).

**Figure 4:**
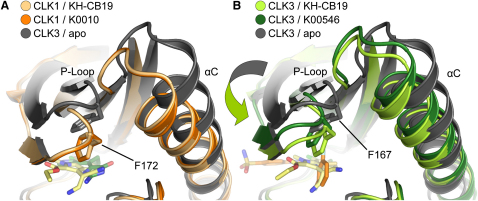
Induced Fit of the P Loop upon Inhibitor Binding Shown are superimpositions of CLK1 (A) and CLK3 (B) inhibitor complexes. The different cocrystallized ligands are indicated in the figure. Inhibitor molecules and the P loop phenylalanine (F172 and F167 in CLK1 and CLK3, respectively) are shown in stick representation. The induced structural changes are indicated by an arrow.

Instead of the canonical polar interactions of ATP-mimetic inhibitors with the kinase hinge, the hydrophilic groups of **KH-CB19** were oriented toward the back of ATP pocket (Figure 3). In particular, the cyano moiety formed a hydrogen bond with the catalytic residue Lys191 (CLK1 numbering), while the amino group made bidentate bonds to the backbone of Glu292 and side chain of Asn293. Cyano moieties that interact with the catalytic lysine are also present in the non-ATP competitive MEK inhibitor U0126, but since this compound does not occupy the ATP site it coordinates the lysine ω-NH_2_ group from the allosteric binding pocket adjacent to the MEK ATP site (Fischmann et al., 2009). The CLK binding geometry packed the N- and C-terminal kinase lobes tightly, making a critical contribution to the overall binding affinity. Another interesting feature of the complex was the wedge-like contact between Phe172 (CLK1) and the inhibitor. Conserved aromatic residues in the Phe172 position on the tip of the phosphate binding loop (P loop) have been proposed to contribute to kinase inhibitor binding (Pogacic et al., 2007; Yamaguchi et al., 2006). For example, the loop dynamics have been postulated to determine kinase isoform selectivity (Doudou et al., 2010). Comparison of the CLK1 and CLK3 structures further supported this hypothesis. The conformation of the P loop was identical in the two CLK1 structures, the complex with **KH-CB19** (Figure 4) and the structurally different and nonselective kinase inhibitor hymenialdisine. The CLK3-**KH-CB19** complex also superimposed well, suggesting that the CLK1 P loop conformation was optimal for **KH-CB19** binding. In contrast, superimposition of the two CLK3 complexes, with **KH-CB19** (Figure 4) and CDK1/2 inhibitor, revealed the preference for CLK3 to adopt a more open conformation with the P loop moving away from the ATP binding site. Therefore, the markedly decreased affinity of **KH-CB19** for CLK3 may reflect the energetic penalty associated with its induced fit.

### Effects of CLK Inhibition on SR Protein Phosphorylation

**Figure 5:**
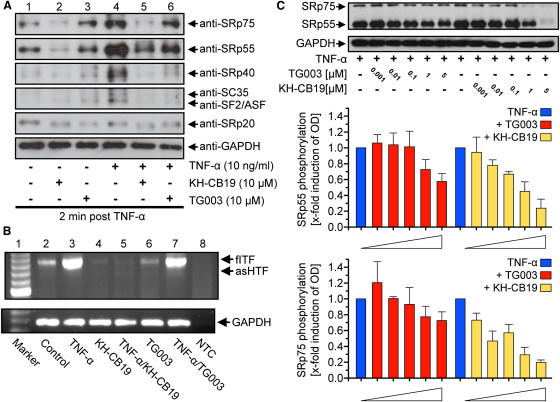
Influence of CLK Inhibitors on the Phosphorylation State of SR Proteins and mRNA Expression of flTF and asHTF in HMEC-1 Cells (A) SR protein phosphorylation is shown in resting or TNF-α-induced HMEC-1 cells 2 min post TNF-α stimulation. SR protein family members SRp75 (75 kDa), SRp55 (55 kDa), SRp40 (40 kDa), SC35 (35 kDa), SF2/ASF (30 kDa), and SRp20 (20 kDa) were detected using phosphorylation-dependent anti-SR protein antibodies. Displayed are nonstimulated cells with or without pretreatment with **KH-CB19** (10 μM) or **TG003** (10 μM) and TNF-α-stimulated cells (10 ng/ml) pretreated with the same CLK inhibitors. The results are representative of at least three independent experiments. (B) Expression of mRNA encoding flTF (931 bp) and asHTF (771 bp) in untreated cells (control), TNF-α−induced HMEC-1 (TNF-α), and cells pretreated with 10 μM compound **KH-CB19** or **TG003** with or without TNF-α 1 hr post stimulation. GAPDH was used as a loading control. A 100 bp DNA ladder was used as a marker. The panel shown is representative of at least three independent experiments. (C) Dose-dependent reduction of phosphorylation of SRp75 and SRp55 and quantitation of the detected phosphorylation (lower panels). GAPDH was used as a loading control. Data were expressed as mean ± SEM using three independent experiments.

To assess the phosphorylation state of SR proteins, western blotting was performed 2 min poststimulation of human microvascular endothelial cells (HMEC-1) with TNF-α (Figure 5A). SRp75, SRp55, SRp40, SC35, SF2/ASF, and SRp20 were detected in HMEC-1 using antibodies that selectively recognize phosphorylated variants of these proteins (Figure 5A, lane 1). Treatment of nonstimulated cells with 10 μM **KH-CB19** led to a reduced phosphorylation of SRp75, SRp55, and SRp20 compared with nonstimulated controls, whereas the phosphorylation of SRp40, SC35, and SF2/ASF was unaffected under basal conditions (lane 2). Pretreatment of HMEC-1 with 10 μM **TG003**, a previously identified CLK inhibitor, only reduced the phosphorylation of SRp20, but had no effect on the phosphorylation state of other SR proteins under normal conditions (lane 3). Stimulation of HMEC-1 with TNF-α led to an increase in the phosphorylation of all detected SR proteins only 2 min post induction (lane 4). Pretreatment of cells with **KH-CB19** or **TG003** led to a reduction of the TNF-α-induced increase in phosphorylation of all analyzed SR proteins (lanes 5 and 6) compared with TNF-α-stimulated controls (lane 3). However, the effect of 10 μM **KH-CB19** was far greater under both normal and proinflammatory conditions as compared to cells treated with 10 μM **TG003**. Dose response of **KH-CB19** was tested using SRp75 and SRp55. As shown in Figure 5C phosphorylation levels of these two proteins in TNF-α−stimulated cells were significantly reduced at increasing concentration of the inhibitor. In contrast, **TG003** had little effect on SRp75 and SRp55 phosphorylation at the tested concentrations.

### Effect of KH-CB19 and TG003 on Alternative Tissue Factor pre-mRNA Splicing in Human Endothelial Cells

Unstimulated HMEC-1 constitutively express both tissue factor (TF) isoforms, the soluble asHTF as well as the membrane bound full-length TF (flTF) at the mRNA level (Figure 5B, lane 2). Stimulation of HMEC-1 with 10 ng/ml TNF-α led to an increased mRNA expression of both TF isoforms compared with nontreated controls (lane 3). Treatment of resting cells with 10 μM **KH-CB19** significantly reduced the basal expression of flTF as well as asHTF (lane 4). Pharmacologic inhibition of CLKs using **KH-CB19** also lowered TNF-α-induced expression of both TF mRNA splice variants to baseline (lane 5). Treatment of HMEC-1 cells with 10 μM **TG003** also reduced the basal expression of flTF and asHTF mRNA (lane 6), and showed only slightly reduced mRNA expression in TNF-α-induced cells 1 hr post stimulation (lane 7).

## Discussion

Despite the substantial effort in developing targeted kinase inhibitors, the task of selective inhibitor design remains highly challenging (Morphy, 2010). As a result, only a handful of reported kinase inhibitors can be classified as truly specific agents (Smyth and Collins, 2009; Karaman et al., 2008). One, albeit not insurmountable challenge, is the overreliance on ATP-mimetic hydrogen bonding to the kinase hinge region. Few kinases have been successfully targeted by other binding mechanisms. A prominent example is PIM1, which has a unique proline residue in the +3 hinge donor position which breaks the classical hydrogen bonding pattern leading to reorientation of inhibitors and formation of polar contacts with the opposing face of the ATP binding pocket (Bullock et al., 2005; Jacobs et al., 2005). These unusual binding modes have been associated with the unique PIM hinge region which does not allow formation of a second hydrogen bond with ATP or ATP mimetic ligands. Here, we report that CDC2-like kinases, which have seemingly nondistinguished and standard sequence around the ATP binding site can be successfully targeted by inhibitors that do not mimic the canonical hydrogen bond pattern of ATP mimetic inhibitors. Crystal structures suggest that this binding mode is optimally satisfied by an inward conformation of the P loop which provides additional interaction through CLK1 Phe172. This work highlights the opportunity to develop very potent and specific inhibitors with new chemical profiles. Comparisons of inhibitor cross reactivity revealed a very favorable selectivity profile for **KH-CB19** when compared with typical ATP mimetic ligands.

An interesting feature of the **KH-CB19** binding mode is the presence of a halogen bond formed with one CLK hinge region backbone carbonyl. Halogen bonds are short-range molecular interactions involving polarized halogens. Such contacts occur frequently in inhibitor target complexes but have only recently been recognized as intermolecular interactions that may favorably contribute to ligand affinity (Hernandes et al., 2010). Well-known examples are halogen bonds that have been described in tetrabromobenzimidazole casein kinase 2 (CK2) complexes (Battistutta et al., 2005). However, more theoretical and experimental studies are needed to understand the biophysical nature of halogen bonding and how these interactions can be exploited in structure based drug design.

Alternative splicing is an essential regulatory process influencing the functional diversity and plasticity of the proteome in response to environmental changes (Black, 2003). CLKs regulate alternative splicing by phosphorylating SR proteins, thereby modulating their cellular localization and splicing activity (Prasad et al., 1999; Tardos et al., 2008; Bourgeois et al., 2004; Eisenreich et al., 2008). The pharmacological inhibition of CLKs is feasible (Muraki et al., 2004) and has been shown to influence alternative splicing of important vascular proteins, such as TF and VEGF (Eisenreich et al., 2009; Nowak et al., 2008). Here, we show that **KH-CB19** suppresses SR protein phosphorylation by CLKs under proinflammatory conditions and that this inhibition is sufficient to modulate the differential expression of the TF isoforms, asHTF and flTF in human endothelial cells. The pharmacologic inhibition of CLKs by **TG003** was also shown previously to reduce the expression of both TF isoforms and to reduce phosphorylation of SRp75, SRp55 and SF2/ASF (Eisenreich et al., 2009). The selectivity of this pharmacologic inhibition to the CLK family was verified by specific siRNA-mediated inhibition of CLK1 as well as CLK4. Here, we demonstrate that the inhibitory effect of **KH-CB19** is more selective and efficacious in cellular assays than inhibitors that have been reported previously (Eisenreich et al., 2009). Thus, **KH-CB19** represents an excellent tool compound for examining the role of CLKs, especially in their regulation of alternative splicing and for further development as a lead compound in drug discovery.

## Significance

**Kinases have been in the focus of drug discovery for more than two decades. Despite the large effort in this target area, only a few highly selective inhibitors have been described. In this study, we identified the dichloroindolyl enaminonitrile KH-CB19 as highly selective inhibitor for CLK kinases. Methylation of the indole nitrogen precluded the canonical ATP mimetic binding mode. Cocrystal structures revealed that hinge interaction of KH-CB19 is mediated by halogen bonding.**

**CLK kinases are key regulators of protein splicing. Consistent with its expected mechanism of action, KH-CB19 effectively suppressed phosphorylation of SR (serine/arginine) splicing factors in cells and significantly altered splicing of the two tissue factor isoforms flTF (full-length TF) and asHTF (alternatively spliced human TF). The discovered inhibitor class is therefore a useful model and an excellent probe compound for the development of inhibitors that target protein splicing. Furthermore, the described binding mode of the discovered dichloroindolyl enaminonitrile inhibitors may serve as a template for the development of selective inhibitors for other kinase targets that explore non-ATP mimetic interactions with the kinase active site and halogen bonding with the hinge backbone.**

## Experimental Procedures

### Protein Expression and Purification

CLK1 and CLK3 were prepared as described (Bullock et al., 2009). In brief, the kinase domains of human CLK1 (residues: 148–484 (C terminus) and CLK3 (residues 275–632) were subcloned by ligation independent cloning into a pET-derived expression vector, pLIC, and expression performed in BL21(DE3) with 1 mM IPTG induction for 4 hr at 18°C. Cells were lysed using a high-pressure homogenizer and cleared by centrifugation and the lysates were purified by Ni-NTA chromatography. The eluted proteins were treated with lambda phosphatase together with TEV protease overnight to remove phosphorylation and the hexahistidine tag, respectively. The proteins were further purified by size exclusion chromatography using a S75 16/60 HiLoad column.

### Thermal Stability Shift Assay

Thermal denaturation experiments were carried out in an Mx3005p real-time PCR machine (Agilent) using a protein concentration of 2 μM and an inhibitor concentration of 10 μM. Samples were buffered in 10 mM HEPES (pH 7.5), 500 mM NaCl and a 1:1000 dilution of SyproOrange (Invitrogen). The assay and data evaluation were carried out as described (Bullock et al., 2005)

### Kinase Inhibition Assay

Phosphorylation reactions were monitored using a coupled-enzyme assay in which ADP production is coupled to NADH oxidation by pyruvate kinase (PK) and lactate dehydrogenase (LDH) as described (Bullock et al., 2009). The reaction was started by addition of 0.1 mM ATP after a 10 min preincubation of the reaction mixture at 25°C. The consensus peptide for CLK1 (AFRREWSPGKEAKK) and the DYRK1A substrate peptide (YRASPSRPESPRPPA-amide) were used as substrates at a concentration of 100 μM. Inhibitors, dissolved in DMSO, were added at the beginning of the preincubation period resulting in a final DMSO concentration of 2% in the assay. Kinetic analysis was performed by nonlinear regression fitting using GraphPad Prism 5 and least-squares fits to sigmoidal dose response curves with variable slope equation:

$$Y=min+\frac{max-min}{1+10^{\left(\log EC50-x\right)Hillslope}},$$

where max and min corresponds to maximal and minimal absorbance value. **K00546** is CDK1/2 inhibitor III purchased from Merck Biosciences (cat. # 217714). **TG003** was purchased from Merck Biosciences (cat. #219479).

Enzymatic kinase selectivity screening was carried out using the Caliper mobility shift assay which is based on the difference in capillary electrophoresis mobility of a fluorescent tagged peptide as a result of the addition of a phosphate moiety by the studied kinase. The kinase reactions were started by addition of 4.5 μl substrate mix consisting of ATP and peptide substrate in assay buffer (50 mM HEPES [pH 7.5], 0.02% bovine serum albumin, 1 mM DTT, 0.02% Tween 20, 0.01 mM Na_3_VO_4_, 10 mM beta-glycerophosphate) and 4.5 μl enzyme solution in assay buffer. The peptide concentration was 2 μM. Concentrations for the enzyme, as well as for MgCl_2_ and MnCl_2_ were adjusted specifically to the requirements of the individual enzyme. ATP concentrations were adjusted to the *K*_M_ values of the specific enzyme. After incubation for 60 min at 30°C the kinase reactions were stopped by addition of 16 μl stop solution (100 mM HEPES [pH 7.5], 5% DMSO, 0.1% coating reagent [Caliper Lifescience] 10 mM EDTA [pH 8.0], 0.015% BRIJ35). Stopped kinase reactions were analyzed in a LC3000 reader (Caliper Lifescience). Compounds were tested from 0.1 nM to 10 μM in eight steps.

### Crystallization

Aliquots of the purified proteins were set up for crystallization using a Mosquito crystallization robot (TTP Labtech, Royston UK). Coarse screens were prepared in Greiner 3-well plates using three different drop ratios of precipitant to protein per condition (100 + 50 nl, 75 + 75 nl, and 50 + 100 nl). Initial hits were optimized using Greiner 1-well plates with an increase in the drop size. All crystallizations were carried out using the sitting drop vapor diffusion method at 4°C. CLK1 crystals with **KH-CB19** (1 mM final concentration) were grown by mixing 100 nl protein (8.0 mg/ml) with 50 nl reservoir solution containing 2.1 M sodium malate pH 7.0. CLK3 crystals with **K00546** or **KH-CB19** were grown by mixing 75 nl of protein (11.4 mg/ml and 1 mM final ligand concentration) with an equal volume of reservoir solution containing 0.2 M (NH_4_)_2_SO_4_, 0.1 M Bis-Tris (pH 5.5), and 25% PEG3350.

### Data Collection and Structure Solution

Crystals were cryoprotected using the well solution supplemented with additional ethylene glycol and were flash frozen in liquid nitrogen in all cases. Data were collected at the Swiss Light Source on beamline X10SA using a MAR225 detector at 1.006029 Å (CLK1/**KH-CB19**), at the Diamond Light Source on beamline I02 using a ADSC Q315 detector at 0.9050 Å (CLK3/**K00546**), or in-house on a Bruker system equipped with a Microstar generator and a Pt135 detector at 1.54 Å. Indexing and integration was carried out using MOSFLM (Leslie and Powell, 2007) (CLK1/**KH-CB19**, CLK3/**K00546**) or XPREP (Sheldrick, 2008) (CLK3/**K01762**) and scaling was performed with SCALA (Evans, 2007). Initial phases were calculated by molecular replacement with PHASER (McCoy et al., 2005) using the known model of CLK1 (PDB ID 1Z57). Initial models were built by ARP/wARP (Perrakis et al., 1999) and building was completed manually with COOT (Emsley and Cowtan, 2004). Refinement was carried out in REFMAC5 (Murshudov et al., 1997) or BUSTER (Bricogne, 1993). In all cases, thermal motions were analyzed using TLSMD (Painter and Merritt, 2006) and hydrogen atoms were included in late refinement cycles. Data collection and refinement statistics can be found in Table 2.

### Chemical Synthesis

#### General Information

NMR spectra were recorded using a Jeol JNMR-GSX 400 and Jeol JNMR-GSX 500 (Jeol, Peabody, MA). *E/Z* ratios were determined by integration of the corresponding peaks in the ^1^H NMR spectra, chemical shifts are given in Hertz. Mass spectra (electronic ionization, EI, 70 eV) were recorded using a Hewlett Packard 5989 A Mass Spectrometer with a 59980 B Particle Beam LC/MS-interface (Agilent Technologies, Palo Alto, CA). High-resolution mass spectra were obtained using a Jeol Mstation 700. Melting points were determined with a Büchi B-540 apparatus (Büchi, Flawil, Switzerland) and are uncorrected. Microwave reactor: CEM Discover (CEM, Matthews, NC). Purification by flash column chromatography (FCC) was done using Silica gel 60 (Merck, Darmstadt, Germany). All solvents and chemicals were purchased from Sigma-Aldrich, Fluka, and Acros.

### (*E/Z*)-Ethyl 6,7-Dichloro-3-[1-Cyano-2-(Dimethylamino)Vinyl]-1-Methyl-1*H*-Indole-2-Carboxylate (2)

Under nitrogen, 2.00 g (11.57 mmol) Bredereck's reagent (*tert*-butoxy-bis(dimethylamino)methane) were added to a solution of 2.00 g (6.43 mmol) ethyl 3-(cyanomethyl)-6,7-dichloro-1-methyl-1*H*-indole-2-carboxylate (**1**) (Huber et al., 2010b) in 10 ml anhydrous DMF and the mixture was stirred at 80°C for 12 hr. The solvent was removed by rotary evaporation and the crude product recrystallized from toluene to give 1.60 g (69%) of **2** as yellow crystals. Mp 154°C; ^1^H NMR (400 MHz, CD_2_Cl_2_, TMS) δ 7.60 (d, *J* = 8.6 Hz, 0.82 × 1 H, 4-H, *Z*), 7.47 (d, *J* = 8.6 Hz, 0.18 × 1 H, 4-H, *E*), 7.27 (d, *J* = 8.6 Hz, 0.18 × 1 H, 5-H, *E*), 7.24 (d, *J* = 8.6 Hz, 0.82 × 1 H, 5-H, *Z*), 6.93 (s, 0.18 × 1 H, 2′-H, *E*), 6.57 (s, 0.82 × 1 H, 2′-H, *Z*), 4.39 (br q, *J* = 7.1 Hz, 2 H, CH_2_), 4.35 (s, 0.18 × 3 H, 1-CH_3_, *E*), 4.28 (s, 0.82 × 3 H, 1-CH_3_, *Z*), 3.21 (s, 6 H, 2′-N(CH_3_)_2_), 1.42 (t, *J* = 7.1 Hz, 3 H, CH_2_CH_3_); ^13^C NMR (100 MHz, CD_2_Cl_2_, TMS) δ 162.0 (C=O, *Z*), 161.7 (C=O, *E*), 153.6 (C-2′, *Z*), 151.4 (C-2′, *E*), 135.2 (C-7a, *Z*), 134.9 (C-7a, *E*), 131.1 (C-6, *E*), 130.8 (C-6, *Z*), 130.5 (C-2, *E*), 129.6 (C-2, *Z*), 129.4 (C-3a, *E*), 128.4 (C-3a, *Z*), 123.7 (CN, *E*), 123.4 (C-5, *E*), 122.9 (C-5, *Z*), 121.5 (CN, *Z*), 120.9 (C-4, *E*), 120.6 (C-4, *Z*), 119.2 (C-3, *Z*), 116.6 (C-7, *E*), 116.5 (C-7, *Z*), 115.7 (C-3, *E*), 68.2 (C-1′, *E*), 65.5 (C-1′, *Z*), 61.9 (CH_2_, *E*), 61.8 (CH_2_, *Z*), 42.5 (2′-N(CH_3_)_2_), 35.6 (1-CH_3_), 14.4 (CH_2_CH_3_); *E/Z* ratio (%) 18:82; MS EI *m/z* (relative intensity, %) 369 [M^+**•**^] (10), 367 [M^+**•**^] (68), 365 [M^+**•**^] (100), 292 (27).

### 7,8-Dichloro-9-Methyl-1-Oxo-2,9-Dihydro-1*H*-Pyrido[3,4-*b*]Indole-4-Carbonitrile (3)

890 mg (2.43 mmol) (*E/Z*)-ethyl 6,7-dichloro-3-[1-cyano-2-(dimethylamino)vinyl]-1-methyl-1*H*-indole-2-carboxylate (**2**), 8.0 g ammonium acetate and 2 ml glacial acetic acid were irradiated in a microwave reactor at 112°C and 150 W power for 45 min. The mixture was poured into ice-water and the precipitate filtered off. The crude product was resuspended in toluene and the solvent removed to give 200 mg (28%) as a beige solid. Mp 410°C (decomp); ^1^H NMR (500 MHz, DMSO-*d_6_*, TMS, 70°C) δ 11.97 (br s, 1 H, N-H), 8.16 (d, *J* = 8.5 Hz, 1 H, 5-H), 8.01 (s, 1 H, 3-H), 7.52 (d, *J* = 8.5 Hz, 1 H, 6-H), 4.61 (s, 3 H, N-CH_3_); ^13^C NMR (500 MHz, DMSO-*d_6_*, TMS, 70°C) δ 155.6 (C=O), 136.6 (C-3), 136.6 (C-8a), 131.7 (C-7), 127.4 (C-9a), 122.9 (C-6), 121.2 (C-4b), 120.1 (C-4a), 120.0 (C-6), 116.7 (CN), 115.9 (C-8), 84.0 (C-4), 34.3 (N-CH_3_); MS EI *m/z* (relative intensity, %) 295 [M^+**•**^] (10), 293 [M^+**•**^] (68), 291 [M^+**•**^] (100).

### (*E/Z*)-Ethyl 3-(2-Amino-1-Cyanovinyl)-6,7-Dichloro-1-Methyl-1*H*-Indole-2-Carboxylate (KH-CB20)

390 mg (1.06 mmol) (*E/Z*)-ethyl 6,7-dichloro-3-[1-cyano-2-(dimethylamino)vinyl]-1-methyl-1*H*-indole-2-carboxylate (**2**), 4.0 g (52 mmol) ammonium acetate, 5 ml glacial acetic acid and 2 ml conc. sulfuric acid were irradiated in a microwave reactor at 112°C and 150 W power for 15 min. After cooling, the mixture was poured into 100 ml cold aqueous ammonia followed by extraction with ethyl acetate (3 × 50 ml). The organic layer was dried over MgSO_4_ and the solvent evaporated. The crude product was purified by FSC (methylene chloride/ethanol 20:1), followed by recrystallization from ethanol to give 260 mg (72%) as yellowish crystals. Mp 390°C (decomp); ^1^H NMR (500 MHz, CD_2_Cl_2_, TMS) δ 7.53 (d, *J* = 8.5 Hz, 0.29 × 1 H, 4-H, *Z*), 7.41 (d, *J* = 8.5 Hz, 0.71 × 1 H, 4-H, *E*), 7.28 (d, *J* = 8.5 Hz, 0.71 × 1 H, 5-H, *E*), 7.24 (d, *J* = 8.5 Hz, 0.29 × 1 H, 5-H, *Z*), 7.17 (t, *J* = 10.7 Hz, 0.71 × 1 H, 2′-H, *E*), 7.05 (t, *J* = 10.7 Hz, 0.29 × 1 H, 2′-H, *Z*), 5.02 (d, *J* = 10.7 Hz, 0.29 × 2 H, NH_2_, *Z*), 4.42 (q, *J* = 7.1 Hz, 0.71 × 2 H, CH_2_, *E*), 4.40 (q, *J* = 7.1 Hz, 0.29 × 2 H, CH_2_, *Z*), 4.39-4.37 (m, 0.71 × 2 H, NH_2_, *E*), 4.38 (s, 0.71 × 3 H, 1-CH_3_, *E*), 4.31 (s, 0.29 × 3 H, 1-CH_3_, *Z*), 1.44 (t, *J* = 7.1 Hz, 0.71 × 3 H, CH_2_CH_3_, *E*), 1.42 (t, *J* = 7.1 Hz, 0.29 × 3 H, CH_2_CH_3_, *Z*); ^13^C NMR (125 MHz, CD_2_Cl_2_, TMS) δ 161.8 (C=O, *Z*), 161.6 (C=O, *E*), 150.4 (C-2′, *Z*), 146.9 (C-2′, *E*), 135.5 (C-7a, *E*), 135.2 (C-7a, *Z*), 131.2 (C-6, *E*), 131.0 (C-6, *Z*), 130.3 (C-2, *E*), 129.5 (C-2, *Z*), 128.2 (C-3a, *Z*), 126.5 (C-3a, *E*), 123.5 (C-5, *E*), 123.1 (C-5, *Z*), 121.5 (CN, *E*), 120.6 (C-4, *E*), 120.3 (C-4, *Z*), 118.2 (CN, *Z*), 116.9 (C-7, *E*), 116.6 (C-7, *Z*), 115.9 (C-3, *Z*), 112.3 (C-3, *E*), 75.0 (C-1′, *E*), 73.0 (C-1′, *Z*), 62.2 (CH_2_, *E*), 61.9 (CH_2_, *Z*), 35.6 (1-CH_3_), 14.4 (CH_2_CH_3_, *Z*), 14.3 (CH_2_CH_3_, *E*); *E/Z* ratio (%) 71:29; MS EI *m/z* (relative intensity, %) 341 [M^+**•**^] (74), 339 [M^+**•**^] (47), 337 [M^+**•**^] (8), 292 (100), 229 (31).

### (*E*)-Ethyl 3-(2-Amino-1-Cyanovinyl)-6,7-Dichloro-1-Methyl-1*H*-Indole-2-Carboxylate (KH-CB19)

Recrystallization of the *E/Z*-mixture **KH-CB20**, obtained by FSC as described above, from toluene gave pure *E*-isomer in 32% yield as yellowish crystals. Mp 410°C (decomp); ^1^H NMR (500 MHz, CD_2_Cl_2_, TMS) δ 7.41 (d, *J* = 8.5 Hz, 1 H, 4-H), 7.28 (d, *J* = 8.5 Hz, 1 H, 5-H), 7.17 (t, *J* = 10.7 Hz, 1 H, 2′-H), 4.46-4.41 (m, 2 H, NH_2_), 4.41 (q, *J* = 7.1 Hz, 2 H, CH_2_), 4.38 (s, 3 H, 1-CH_3_), 1.44 (t, *J* = 7.1 Hz, 3 H, CH_2_CH_3_); ^13^C NMR (125 MHz, CD_2_Cl_2_, TMS) δ 161.6 (C=O), 146.9 (C-2′), 135.5 (C-7a), 131.1 (C-6), 130.2 (C-2), 126.5 (C-3a), 123.5 (C-5), 121.6 (CN), 120.6 (C-4), 116.9 (C-7), 112.3 (C-3), 74.9 (C-1′), 62.2 (CH_2_), 35.6 (1-CH_3_), 14.3 (CH_2_CH_3_); MS EI *m/z* (relative intensity, %) 341 [M^+**•**^] (7), 339 [M^+**•**^] (50), 337 [M^+**•**^] (69), 292 (100), 229 (32), 149 (64).

### Cell Culture

Human microvascular cells (HMEC-1) were cultured in endothelial cell (EC) growth medium containing 5% fetal calf serum at 37°C in a humidified incubator (5% CO_2_, 95% air). Cells from passages 2 to 6 were used. For inhibition experiments, HMEC-1 endothelial cells were switched to EC basal medium (without fetal calf serum) for 1 hr. After that, cells were pretreated with the CLK inhibitor **KH-CB19** (1 nM to 100 μM) or **TG003** (10 μM; Calbiochem, Darmstadt, Germany), respectively, for 1 hr and then stimulated with 10 ng/ml TNF-α (Sigma Aldrich, St Louis, MO). Analysis of the TF isoform mRNA expression was done 1 hr post stimulation with TNF-α and assessment of the phosphorylation state of SR proteins by western blotting was performed 2 min post induction of the cells. Positive controls were stimulated only with TNF-α, and negative controls were untreated.

### TF Isoform-Specific Real-Time RT-PCR

Real-time PCR employing flTF, asHTF, and GAPDH-specific primers and probes was performed as described previously (Szotowski et al., 2005).

### Semiquantitative RT-PCR

For semiquantitative RT-PCR the following primers were used: hTF_left_1 (5′-CGC CGCCAACTGGTAGAC-3′), hTF_right_1 (5′-TGCAGTAGCTCCAACAGTGC-3′), GAPDH_For (5′-GAGTCAACGGATTTGGTCGT-3′) and GAPDH_Rev (5′-GACAAG CTTCCCGTTCTCAG-3′). PCR conditions were as follows: 94°C, 2 min, and 36 cycles of 94°C, 30 sec; 58°C, 30 sec; and 72°C, 1 min. PCR products were separated on 1.5% agarose gels, excised, and purified and their identity was confirmed by automated sequencing.

### Western Blotting

Western blot analysis of samples from cell lysates of inhibited, stimulated, and unstimulated HMEC-1 cells were performed as previously described (Szotowski et al., 2005). For detection of phosphorylated SR proteins, monoclonal antibody mAb1H4 (Invitrogen GmbH, Karlsruhe, Germany) was used.

### Statistical Analysis

All data were expressed as mean ± SEM. Data were analyzed by Student's t test or 1-way ANOVA. A probability value ≤ 0.05 was deemed significant.

## Figures and Tables

**Table 1:** Effect of the Studied Inhibitors on Enzymatic Activity

Inhibitor	CLK1 [nM]	CLK3 [nM]	DYRK1A [nM]
KH-CB19	19.7 ± 6	530 ± 140	55.2 ± 6
KH-CB20	16.5 ± 3	488 ± 120	57.8 ± 2
TG003	48.6 ± 16	>4000	156.1 ± 23.0
K00546	8.9 ± 3	29.2 ± 8	ND

IC_50_ values are shown in nM and values were average from three independent experiments. Literature values for TG003 according to Muraki et al. (2004): CLK1, 20 nM, CLK4 15 nM, CLK3 >10 μM. ND, not determined.

**Table 2:** Data Collection and Refinement Statistics

Data Collection			
PDB ID	2VAG	2WU6	2WU7
Target	CLK1	CLK3	CLK3
Inhibitor	**KH-CB19**	**K00546**	**KH-CB19**
Space group	C2	C2	C2
Cell dimensions: a, b, c (Å)	90.95, 64.11, 78.89	89.15, 62.33, 74.15	87.58, 62.03, 75.08
α, β, γ (deg)	90.00, 118.17, 90.00	90.0, 96.04, 90.0	90.0, 98.05, 90.0
Resolution^a^ (Å)	1.80 (1.92–1.80)	1.92 (2.02–1.92)	2.23 (2.28–2.23)
Unique observations^a^	36,979 (5380)	30,158 (4343)	19,441(861)
Completeness^a^ (%)	99.9 (99.9)	97.1 (96.2)	99.9 (97.8)
Redundancy^a^	3.8 (3.0)	4.3 (4.4)	6.59 (4.45)
R_merge_^a^	0.090 (0.613)	0.132 (0.761)	0.058 (0.581)
I/ σI^a^	11.6 (1.8)	7.3 (2.0)	7.26 (2.1)
Refinement			
Resolution (Å)	1.80	1.92	2.23
R_work_ / R_free_ (%)	18.2/22.5	16.9/21.4	19.1/27.2
Number of atoms(protein/other/water)	2645/22/230	2853/55/305	2796/52/110
B factors (Å^2^)(protein/other/water)	20.66/13.71/25.45	23.80/23.38/21.08	17.70/25.46/8.66
Rmsd bonds (Å)	0.013	0.016	0.016
Rmsd angles (^o^)	1.404	1.555	1.570
Ramachandran favored (%)	96.04	97.09	96.24
Allowed (%)	2.74	2.33	2.31
Disallowed (%)	1.22	0.58	1.45

a Values in parentheses correspond to the highest resolution shell.

## Accession Numbers

The models and structure factors have been deposited with PDB accession codes 2VAG (CLK1/**KH-CB19**), 2WU6 (CLK3/**K00546**), and 2WU7 (CLK3/**KH-CB19**).

## Supplemental Information

Document S1. One Table
