# Supplementary material for: Specific CLK Inhibitors from a Novel Chemotype for Regulation of Alternative Splicing
Source: Chem Biol. 2011 Jan 28;18(1):67–76. doi: 10.1016/j.chembiol.2010.11.009 (PMC3145970; doi:10.1016/j.chembiol.2010.11.009)
Supplement: Document S1. One Table [file mmc1.pdf]

Chemistry & Biology ***18***

Supplemental Information

Specific CLK INHIBITORS

from a Novel Chemotype

for Regulation of Alternative Splicing

Oleg Fedorov, Kilian Huber, Andreas Eisenreich, Panagis Filippakopoulos, Oliver King, Alex N. Bullock, Damian Szklarczyk, Lars J. Jensen, Doriano Fabbro, Jörg Trappe, Ursula Rauch, Franz Bracher, and Stefan Knapp

Table S1, related to Figure 2. Effect of inhibitors on protein thermal stability

Kinase	Hymenialdisine (K00010)	K00546	KH-CB19	KH-CB20	3	2	TG003
AAK1	2.7	9.8	1.3	1.3	1.4	0.3	1.6
ACTR2	0.7	6.3	0.2	0.4	0.8	0.6	-0.3
AKT3	1.3	0.0	-0.2	-0.3	0.1	-0.2	0.1
ALK2	2.1	12.5	0.5	0.8	0.5	-0.5	1.2
ALK4	4.9	10.3	1.2	1.4	0.9	4.0	0.1
ARG		7.6					-0.3
BARK2	-0.3	-0.3	-0.4	-0.1	0.3	0.2	-0.2
BIKE	7.0	14.3			2.7	0.4	0.2
BMPR2	9.9	12.1			0.6	-0.1	0.1
BMX	-0.1	3.1	0.2	0.2	-0.1	-0.2	-0.1
BRK	0.1	2.9			0.0		0.0
CaMK1d	0.0	3.2	0.1	-0.6	-0.3	-0.4	0.1
CaMK1g	-0.2	2.0					-0.5
CaMK2a	0.4	3.3	0.5	0.7	0.5	1.0	-0.1
CaMK2b	0.4	1.6					0.3
CaMK2d	0.0	0.7	0.3	0.1		-0.1	-0.6
CaMK2g	0.2	4.2					0.1
CaMK4	0.4	5.9	0.8	-0.1	0.4	0.2	-0.2
CaMKK1	0.2	4.0	-0.1	0.6	0.4	0.3	0.0
CaMKK2	3.4	8.6			0.5		0.4
CDK2	1.5	16.1	0.2	0.2	0.4	-0.1	0.2
CDK4		4.7					-0.6
CDK6	1.1	4.6					0.4
CDK8	0.1	1.3	1.5	1.3	2.5	0.4	0.1
CDKL1	0.6	4.3	0.0	0.2	0.2	0.6	0.4
ChaK1	0.3	0.6					-0.1
CHK2	5.7	8.7	0.4	1.0	0.5	0.3	0.1
CK1e	4.8	3.1	0.8	1.0	0.9	0.3	5.1
CK1g1	5.7	7.3					2.5
CK1g2	4.4	4.9					2.7
CK1g3	3.5	5.3	0.1	0.3	0.0	0.5	3.3
CK2a1	0.8	2.4	1.3	1.1	1.3	1.4	2.6
CK2a2	1.2	2.4	1.4	1.3	1.7	0.0	2.6
CLK1	7.8	11.5	10.9	10.2	10.9	5.6	6.7
CLK2	4.4	10.5	4.7	4.8	6.3	3.0	5.0

CLK3	7.2	14.7	6.9	7.0	7.9	1.7	2.5
CLK4	7.0	12.2	12.5	10.8	9.6	4.1	9.8
DAPK3	7.6	3.0	2.2	2.2	2.0	0.3	0.9
DCAMKL1	0.5	5.4	-0.2	-0.1	0.0	0.7	-0.5
DMPK1	0.2	5.4	-0.4	-0.4	0.9	-0.3	1.0
DMPK2	-0.7	0.3			0.7		-1.3
DRAK1	14.8	12.6	2.5	2.6	1.7	-0.2	1.7
DRAK2	13.0	14.8	1.7	1.5	0.9	0.0	3.2
DYRK1A	4.4	2.6	5.4	5.4	5.1	0.6	4.4
DYRK2	0.8	4.3	1.0	0.9	2.1	0.7	7.3
eEF2K	-0.1	-0.1	-0.1	-0.1	0.3	-0.4	-0.3
Erk1	1.1	3.6	-0.3	0.1	-0.5	0.1	-0.4
Erk3	-0.1	2.1					0.0
FES	0.7	3.5	-0.3	-0.3	0.0	0.0	-0.1
FGR	0.7	3.5	-0.3	0.3	0.1	-0.2	-0.2
GAK	3.8	0.4	0.4	0.8	0.8	0.4	0.6
GPRK5	1.3	2.0	0.4	0.2		0.3	0.4
GSK3B	7.4	8.0			5.9	2.7	1.4
Haspin	3.9	0.6	2.1	1.8	2.1	0.2	4.8
IKKb	0.7	5.7	-0.1	0.1	0.5	0.6	0.0
ITK	0.0	3.9					1.1
JAK1	0.3	9.7	0.7	1.0	0.9	0.2	0.0
JNK1	-0.5	4.0	0.9	0.2	0.2	0.1	0.9
JNK2	-0.1	1.8	-0.2	0.5	-0.5	-0.2	0.4
LIMK1	0.4						
LOK	4.2	15.5	-0.8	3.0	-0.7	-1.1	0.7
LYN	0.3	5.3	0.2	0.0	0.2	0.0	0.1
MAP2K2	2.0	5.1	0.1	0.0	0.1	-0.4	0.5
MAP2K6	0.6	7.1	0.5	0.6	0.1	0.4	0.0
MAP3K5	8.7	16.8	0.8	0.5	0.8	0.2	0.4
MER	0.3	3.8			0.9	-0.3	0.0
MPSK1	0.7	7.8					0.4
MSSK1	0.3	4.7	0.2	0.5	0.3	0.3	0.0
MST1	4.0	12.9			-0.2		0.7
MST2	3.9	12.4	0.2	0.4	-0.5	0.3	0.1
MST3	1.0	10.5	0.2	-0.1	0.1	-1.1	-0.3
MST4	4.6	11.1	2.4	0.1	0.4	-0.6	-0.3
MYT1	0.6	0.0					-0.8
NDR1	1.0	7.3	0.2	0.3	0.7	0.4	0.3
NDR2	0.9	10.5	-0.2	-0.3	0.0	-0.1	0.3
NEK2	0.8	2.5			0.1		0.8
NEK6	0.0	0.2					-1.0
NEK7	0.6	1.4	0.0	0.1	0.6	-0.7	-0.5
OSR1	0.5	5.7	0.3	0.3	0.3	0.0	0.1
p38b	0.8	1.8			-0.8	-0.6	0.0
p38d	-0.1	2.9	0.3	-0.3	0.3	0.4	0.7
PAK2		-0.4					-0.6
PAK4		7.3					1.7
PAK4	0.3	6.9	0.3	0.2	-0.1		0.4
PAK5	0.2	5.6			0.1		-0.1
PAK6	0.3	5.6					-0.9
PBK	-0.4	1.5	-0.3	-0.1	-0.4	-0.9	0.6
PCTAIRE1	0.7	9.8	0.4	0.1	0.4	0.4	-0.4

PCTAIRE2	0.2	5.1	-0.3	-0.2	0.0		0.0
PDHK1	-0.2	0.4					0.2
PHKg2	1.6	4.6					0.2
PIM1	0.6	1.0	2.9	3.0	3.4	2.5	6.0
PIM2	3.5	0.6	2.1	2.1	3.0	0.8	3.5
PIM3	3.8	1.8	3.1	2.8	4.2	3.2	4.9
PKACa	0.8	4.1	0.0	0.3	0.4	0.2	-0.2
PKCz	-0.2	4.9	-0.2	0.1	0.5	0.4	-0.2
PKD2	0.8	2.2	0.1	-0.2	0.1	0.0	0.7
PKD3	2.0	4.3	0.1	0.3	0.4	0.0	2.6
PKG1	1.4	2.4	0.8	0.5	0.6	0.3	1.6
PKG2	-0.5	-0.5					-0.1
PKN1	1.3	6.0			1.2		-0.5
PKN2	0.8	11.9	0.6	0.1	2.3	-0.5	-0.5
PLK1		4.3	0.3	0.6	0.7	0.9	0.3
PLK4	3.0	13.1	0.7	0.2	0.5	0.2	0.3
PRKX	0.3	3.4	0.5	0.4	0.5	0.3	-0.2
RHOK	0.7	1.2	0.5	0.3	0.9	0.9	0.4
RIOK2	0.2	-0.4					-0.5
RSK1~N		8.6					0.3
RSK1-C	0.7	4.0	1.6	1.9	1.9	0.3	0.9
RSK2~C	2.7	12.3	2.0	1.0	1.3	-0.2	0.4
RSK2~N	0.8	10.5					0.7
RSK3~C	4.5	8.9	0.7	0.4	0.5	0.1	0.4
RSK3~N	4.2	9.7			0.1	0.3	-0.2
RSK4	1.7	8.5	1.6	1.1	1.3	-0.2	0.5
SgK085	2.7	4.5	4.1	4.5	2.9	0.4	0.9
SIK	0.1	3.8					1.5
SLK	2.1	12.1	0.0	0.1	-0.6	-0.7	-0.6
smMLCK	1.9	1.8	0.5	0.6	0.5	1.1	-0.2
SRPK1	0.3	8.2	-0.5	-0.5	-0.5		0.0
SRPK2	-0.2	4.0	0.1	0.5	0.2	-0.1	-0.1
STK33	3.9	5.7	0.3	0.8	1.1	0.7	0.6
STLK3	1.9	8.3					0.5
TEC	0.4	3.7					1.0
TGFbR1	5.7	10.6					0.1
TGFbR2	7.1	7.6			5.7		1.8
TLK1	1.0	4.2					-0.1
TTK	0.7	8.0	0.5	0.3	1.8	-0.1	1.1
TYRO3	0.4	2.8			-0.1	-1.0	0.1
VRK1	2.0	1.9	0.0	0.2	-0.4	0.2	1.1
VRK2	0.8	2.0	-0.1	0.1	0.1	0.2	0.3
VRK3	0.3	1.0			-0.2	0.1	0.5
YANK1	0.0	4.3	-0.2	-0.3	0.3	0.0	-0.2
YANK3	-0.5	1.8	0.2	-0.2		0.4	-0.9
YSK1	1.7	12.6	-0.1	0.1	0.2	0.6	0.6
ZAK	1.7	1.4	-0.3	-0.1	-0.2	-0.4	-0.6
ZC2/TNIK	3.4	9.3	0.2	-0.1	0.0	0.1	0.1

Values are shown in ^{o}C. Empty space indicated that no data have been measured. Targets with significant Tm shift have been colored in red (>9 degrees), orange (6-9 degrees) and yellow (3-6 degrees). Screening concentration was 10μM.
